# Detection of Mixed Infections with *Plasmodium* spp. by PCR, India, 2014

**DOI:** 10.3201/eid2110.150678

**Published:** 2015-10

**Authors:** Sri Krishna, Praveen K. Bharti, Himashu S. Chandel, Amreen Ahmad, Rajesh Kumar, Puspendra P. Singh, Mrigendra P. Singh, Neeru Singh

**Affiliations:** National Institute for Research in Tribal Health, Jabalpur, India (S. Krishna, P.K. Bharti, H.S. Chandel, A. Ahmad, R. Kumar, P.P. Singh, N. Singh);; National Institute for Malaria Research Field Unit, Jabalpur (M.P. Singh)

**Keywords:** Plasmodium falciparum, Plasmodium spp., malaria, parasites, mixed infections, PCR, India

## Abstract

In 8 malaria-endemic states in India, mixed *Plasmodium* spp. infections were detected by PCR in 17.4% (265/1,521) of blood samples that microscopy had shown to contain only *P. falciparum*. The quality of microscopy must be improved because use of PCR for detection of malaria parasites is limited in rural areas.

Five *Plasmodium* species (*P. falciparum*, *P. vivax*, *P. malariae*, *P. ovale*, and *P. knowlesi*) cause human malaria. Malaria is not uniformly distributed in India; 8 of the 35 states and union territories contain most malaria cases (*1*). Infections with *P. falciparum* and *P. vivax* occur at approximately equal frequencies (*2**–**4*). This finding increases the possibility of mixed infections, as reported in other countries, such as Peru (*5*), Papua New Guinea (*6*), Brazil (*7*), and Ethiopia (*8*).

In India, malaria control usually involves vector control with indoor residual spraying of insecticides and insecticide-treated bed nets, and chemotherapy with artemisinin-based combination therapy. Malaria diagnosis is based mainly on microscopic detection of parasites in peripheral blood smears from symptomatic persons. In addition, bivalent, rapid diagnostic tests (RDTs) are useful detection tools (*9*) but cannot differentiate *P. falciparum* moninfections from co-infections with other *Plasmodium* species (*2**,**3*). Moreover, genetic polymorphisms in diagnostic antigens limits detection by monoclonal antibodies. Misdiagnoses might also arise from gene deletions that prevent expression of proteins by the parasite (*10*). We report that a high proportion of mixed infections with 4 *Plasmodium* species detected by PCR in 8 states of India to which malaria is highly endemic were not detected by bivalent RDTs and microscopy.

## The Study

This study was approved by the Institutional review board of the National Institute for Research in Tribal Health (Jabalpur, India). Written informed consent was obtained from all participants or parents of children, according to Indian Council of Medical Research guidelines.

**Figure 1:**
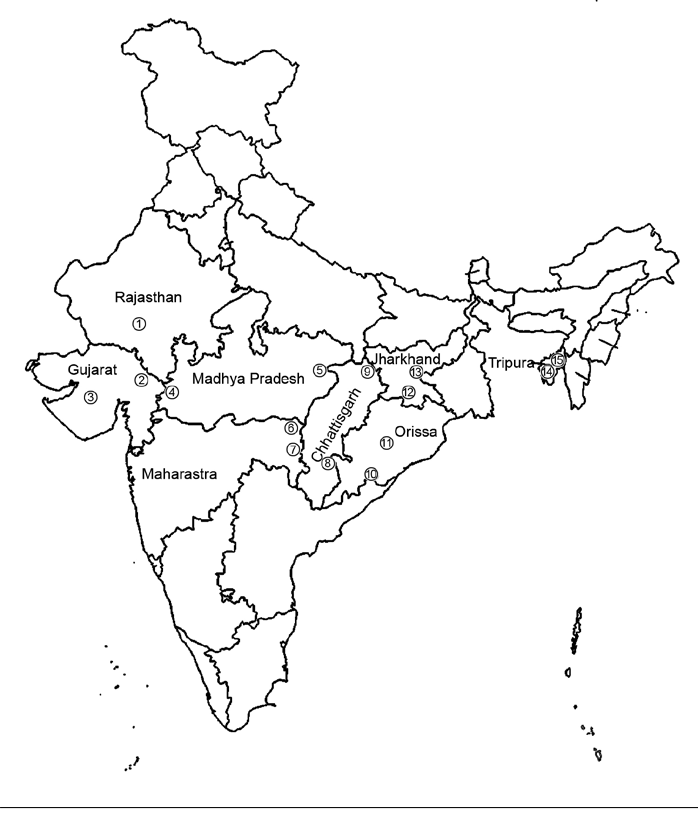
Fifteen community health centers in 8 states in India to which malaria is endemic. 1, Udaipur; 2, Dahod; 3, Valsad; 4, Jhabua; 5, Annupur; 6, Gondia; 7, Gadchiroli; 8, Jagdalpur; 9, Baikunthpur; 10, Koraput; 11, Rayagada; 12, Jaldega; 13, Bano; 14, Manu Bazar; 15, Shantir Bazar.

The study was conducted in 2 community health centers (CHCs), 1 in an area that had a high level of malaria endemicity and 1 that had a low level of malaria endemicity, in each of 8 states in India: Orissa, Chhattisgarh, Jharkhand, Maharashtra, Madhya Pradesh, Tripura, Gujarat, and Rajasthan (Figure 1; Table 1). Selected CHCs were located in different regions, and forest areas in these regions ranged from 13% in Jhabua (Madhya Pradesh) to 81% in Tripura. Elevation above sea level ranged from 13 m in Valsad (Gujarat) to 870 m in Koraput (Orissa). Inhabitants of most study areas were ethnic tribes (39%–87%). All areas had received 2 rounds of indoor residual spray (DDT/synthetic pyrethroid) as a vector control measure.

**Table 1:** Characteristics of mixed infections with 4 *Plasmodium* species identified by PCR for 1,521 blood samples that were *P. falciparum*–positive by microscopy in 8 malaria-endemic states, by district, India, 2014*

District (state) or variable	CHC	Period	No. infections					Odds ratio (95% CI), p value†	
			*Pf*	*Pf* + *Pv*	*Pf* + *Pm*	*Pf* + *Po*	*Pf* + *Pm* + *Po*	Mixed (*Pf* + *Pv*)	Mixed (all pooled)
Koraput (OD)	Bandhugaon	Jul–Aug	188	35	4	2	0	–	–
Rayagada (OD)	Jagannathpur	Jul–Aug	31	5	2	0	0	1.8 (1.0–3.1), 0.004	2.0 (1.2–3.4), 0.0091
Simdega (JH)	Jaldega	Aug–Nov	82	41	0	2	0	–	–
Simdega (JH)	Bano	Aug– Nov	76	14	0	0	1	3.5 (2.0–6.0), <0.0001	3.4 (2.0–5.8), <0.0001
Jagdalpur (CG)	Maharani Hospital	Jul–Oct	178	23	2	1	0	–	–
Baikunthpur (CG)	District Hospital	Jul–Nov	10	0	0	0	0	1.2 (0.7–2.3), 0.5292	1.3 (0.7–2.3), 0.4324
Jhabua (MP)	Ranapur	Sep–Oct	92	29	3	1	0	–	–
Anuppur (MP)	Pushprajgarh	Sep–Nov	82	18	1	0	0	2.7 (1.5–4.7), 0.0004	2.7 (1.6–4.7), 0.0001
Gadchiroli (MH)	Malewada	Sep–Nov	108	6	0	0	0	–	–
Gondia (MH)	Darekasa	Sep–Nov	103	15	2	0	0	1 (Ref)	1 (Ref)
Udaipur (RJ)	Bekaria	Sep–Dec	112	26	2	0	0	2.3 (1.2–4.3), 0.0068	2.3 (1.3–4.2), 0.0056
Dahod (GJ)	Devgadh Baria	Sep–Dec	77	8	2	0	0	–	–
Valsad (GJ)	Lavkar	Sep–Nov	10	0	0	0	0	0.9 (0.4–2.1), 0.8316	1.1 (0.5–2.3), 0.8946
South Tripura (TR)	Manubazar	Oct–Dec	41	4	0	0	0	–	–
South Tripura (TR)	Santirbazar	Oct–Dec	66	15	1	0	0	1.8 (0.9–3.5), 0.084	1.7 (0.9–3.3), 0.0978
Total	NA	NA	1,256	239	19	6	1	NA	NA
Median no. parasites/µL	NA	NA	1,897.3	1,600	1,273.6	1,180	4,040	NA	NA
Range	NA	NA	35–1,785,714	40–380,464	31–56,818	200–124,118	NA	NA	NA
p value	NA	NA	Ref	0.138	0.251	0.439	NA	NA	NA

*CHC, community health center; *Pf*, *P. falciparum*; *Pv*, *P. vivax*; *Pm*, *P. malariae*; *Po*, *P. ovale*; OD, Odisha; JH, Jharkhand; CG, Chhattisgarh; MP, Madhya Pradesh; MH, Maharashtra; Ref, reference; RJ, Rajasthan; GJ, Gujrat; TR, Tripura; NA, not applicable. †–, indicates that analysis was conducted after data for the 2 districts in that state were combined.

Blood samples were collected from persons with suspected malaria during July–December 2014 at malaria clinics in CHC hospitals at 15 sites. Microscopy and RDTs (Bioline Ag Malaria Pf/Pv Test; Standard Diagnostics Inc., Gyeonggi-do, South Korea) were performed at outpatient department clinics of CHCs, and molecular diagnosis (PCR and sequencing) was performed at the molecular parasitology laboratory at the National Institute for Research in Tribal Health.

For microscopy, thick and thin blood smears were prepared from finger prick blood samples, which were air-dried, fixed in methanol, and stained with Giemsa. A total of 100 thick blood smear fields were examined by using an oil immersion lens at 100× magnification before a sample was considered negative. Malaria parasite density was determined from thick blood smears by counting the number of parasites against 200 leukocytes (*11*). Microscopy was also performed on samples that had negative results by RDT. Blood smears were cross-checked by a senior laboratory technician. RDT was performed according to manufacturer’s instructions (*9*) and was repeated for samples in which discordant results were obtained (e.g., microscopy positive, RDT negative).

Genomic DNA was isolated from samples that microscopy showed to contain only *P.* *falciparum* by using the QIAamp DNA Blood Mini Kit (QIAGEN, Hilden, Germany). Species-specific nested PCRs that targeted the 18S rRNA gene were used to detect 4 malaria parasite species (*P. falciparum*, *P. vivax*, *P. ovale*, and *P. malariae*) (*12*). *P. knowlesi* was detected by using a set of primers specific for the 18S rRNA gene (*13*), and differentiation of 2 subspecies of *P. ovale* (*P. curtisi* and *P.* *wallikeri*) was performed as described (*4*). PCR primers and conditions are shown in Table 2. An independent research assistant, who was unaware of microscopy or RDTs results, performed PCR on coded samples.

**Table 2:** Characteristics of PCR primers specific for 18S rRNA gene of *Plasmodium* spp., India*

Genus or species	Primer	Sequence, 5′→3′	PCR product, bp	PCR Program						No. cycles
Denaturation		Annealing		Elongation	
Temp, °C	Time, min	Temp, °C	Time, min	Temp ,°C	Time, min
*Plasmodium*	F	TTAAAATTGTTGCAGTTAAAACG	1,200	94	1	58	2	72	2	25
	R	CCTGTTGTTGCCTTAAACTTC	1,200	94	1	58	2	72	2	25
*P. falciparum*	F	TTAAACTGGTTTGGGAAAACCAAATATATT	205	94	1	58	2	72	2	30
	R	ACACAATGAACTCAATCATGACTACCCGTC	205	94	1	58	2	72	2	30
*P. vivax*	F	CGCTTCTAGCTTAATCCACATAACTGATAC	120	94	1	58	2	72	2	30
	R	ACTTCCAAGCCGAAGCAAAGAAAG TCCTTA	120	94	1	58	2	72	2	30
*P. malariae*	F	ATAACATAGTTGTACGTTAAGAATAACCGC	144	94	1	58	2	72	2	30
	R	AAAATTCCCATGCATAAAAAATTATACAAA	144	94	1	58	2	72	2	30
*P. ovale*	F	ATCTCTTTTGCTATTTTTTAGTATTGGAGA	800	94	1	58	2	72	2	30
	R	GGAAAAGGACACATTAATTGTATCCTAGTG	800	94	1	58	2	72	2	30
*P. knowlesi*	F	CAGAGATCCGTTCTCATGATTTCCATGG	209	95	0.5	57	0.5	72	0.75	35
	R	CTRAACACCTCATGTCGTGGTAG	209	95	0.5	57	0.5	72	0.75	35
*P. ovale*	F	CTACTTGACATTTCTACTTACA	938	95	1	50	1	72	1	35
	R	CGTTCTTGATTAATGGAAGTAT	938	95	1	50	1	72	1	35
*P. ovale* (*curtisi* and *wallikeri*)	F	GCTGTAGCTAATACTTGCTTTA	827	95	1	55	1	72	1	25
	R	TTCACCTCTGACATCTGAATC	827	95	1	55	1	72	1	25

*F, forward; R, reverse.

**Figure 2:**
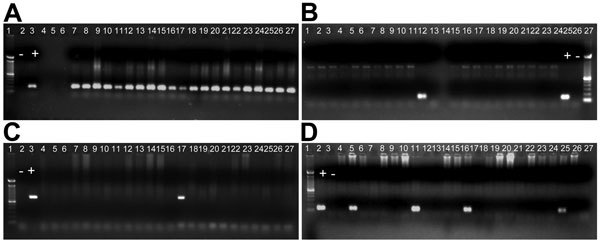
Identification of *Plasmodium* spp. by nested PCR at 15 community health centers in 8 states in India to which malaria is endemic. A) *Plasmodium falciparum* (205-bp fragment). Lane 1, molecular mass marker; lane 2, negative (–) control; lane 3, positive (+) control; lanes 7−27, positive samples; lanes 5 and 6, negative samples. B) *P. malariae* (144-bp fragment). Lane 25, + control; lane 26, – control; lane 27, molecular mass marker; lane 12, positive sample; lanes 1–11, 13–24, negative samples. C) *P. ovale* (800-bp fragment). Lane 1, molecular mass marker; lane 2, – control; lane 3, + control; lane 17, positive sample; lanes 4–16, 18–27, negative samples. D) *P. vivax* (120-bp fragment). Lane 1, molecular mass marker; lane 2, + control; lane 3, – control; lanes 5, 11, 16, and 25, positive samples; lanes 4, 6–10, 12–15, 17–24, 26, and 27, negative samples.

Of 1,521 samples determined by microscopy to be *P. falciparum* moninfections, PCR confirmed results for 1,256 (83%). However, PCR showed mixed infections with *P. falciparum* and *P. vivax* in 239 (16%) samples; *P. falciparum* and *P. malariae* in 19 (1%) samples; *P. falciparum* and *P. ovale* in 6 (0.4%) samples; and *P.* *falciparum*, *P. malariae*, and *P. ovale* in 1 (0.1%) sample (Table 1). Microscopy could not identify these mixed infections (17.4% [265/1,521]). PCR amplification of DNA from 4 *Plasmodium* species is shown in Figure 2.

Secondary microscopic analysis of blood smears by a second technician showed that only 22/239 (9.2%) samples contained mixed infections with *P. falciparum* and *P. vivax*. PCR analysis showed that the highest prevalence of mixed infections with *P. falciparum* and *P. vivax* was in Jharkhand (25.5%, 55/216), followed by Madhya Pradesh (20.8%, 47/226), Rajasthan (18.6%, 26/140), Orissa (15%, 40/267), and Tripura (15%, 19/127), and Chhattisgarh (10.7%, 23/214). The lowest prevalences were in Maharashtra (9%, 21/234) and Gujarat (8.2%, 8/97).

Mixed infections with *P. falciparum* and *P. malariae* were found in all 8 states, although in small numbers. Mixed infections with *P. falciparum* and *P. ovale* were found in only 4 states, particularly at CHCs in areas to which malaria was highly endemic. Of 7 mixed infections that contained *P. ovale*, 5 contained *P. ovale* *curtisi* and 2 contained *P. ovale* *wallikeri*. *P. knowlesi* was not found in any state.

## Conclusions

This study was conducted 8 states in India that contain 80% of malaria cases (85% of which are caused by *P. falciparum*) and 70% of deaths caused by malaria in the entire country (*1*). Misdiagnosis by microscopy occurs because in mixed infections there is a tendency of 1 parasite to predominate and microscopy usually does not detect low numbers of other parasites (*6*). Thus, rare malaria parasites and mixed infections are underestimated through routine microscopy and RDTs (*2**–**6*), and misidentification of malaria parasites could prolong parasite clearance time and lead to anemia and drug resistance (*14*). A high proportion of mixed infections with *P. vivax* and *P. falciparum* have been reported in India (*15*). However, in that study, Gupta et al. did not look for *P. malariae* or *P. ovale* and their sample size was small (180 persons).

Our study had some limitations. Monoinfections or mixed infections were not verified by PCR if parasitemia levels were too low to be detected by microscopy or RDTs. Thus, mixed infections with parasitemia levels below the limit of detection of microscopy or RDTs would not have been detected. Detailed studies in different ecosystems during different transmission seasons and large sample sizes are required for a more accurate picture of mixed infections with common and uncommon parasite species, clinical epidemiology, adverse effects, relapse, and recrudescence.

Our results highlight the role of mixed infections, particularly those with *P. vivax*, *P.* *malariae*, and *P. ovale*, which are not detected accurately by microscopy or RDTs. Although *P.* *vivax* and *P. ovale* are responsible for relapses (*4*), *P. malariae* is sustained at low rates among sparse and mobile human populations for decades, thus facilitating transmission by mosquitoes (*2*). Our results also emphasize a major concern in the diagnosis of malaria by microscopy or RDTs and has serious repercussions for malaria epidemiology and subsequent control. These findings indicate the need to improve quality of microscopy and RDTs because PCR techniques are expensive. Until PCR becomes much less expensive and more available as a point-of-care test for field testing, its use will be limited for malaria detection.
